# Predictability of thermo-lesions using electrodes for deep brain stimulation - an in vitro study

**DOI:** 10.1186/1756-0500-3-84

**Published:** 2010-03-26

**Authors:** Thomas L Hauska, Hermann Lanmüller, Wolfgang Kainz, François Alesch

**Affiliations:** 1 Department of Neurosurgery, Medical University Vienna, Währinger Gürtel 18-20, 1090 Vienna, Austria; 2 Center for Biomedical Technics and Physics, Medical University Vienna, Währinger Gürtel 18-20, 1090 Vienna, Austria; 3 Center for Devices and Radiological Health, FDA, 10903 New Hampshire Avenue, Silver Spring, MD 20993, USA

## Abstract

**Background:**

Typically, electrodes for Deep Brain Stimulation (DBS) are used for chronic stimulation. However, there are conditions where this therapy has to be discontinued. In such cases using the DBS electrodes as a tool for thermo-lesioning (coagulation) could be used for an alternative treatment. The aim of this study was to determine if it is possible to generate coagula with a predictable geometry and to define their dimensions as a function of power and time in an in vitro model (egg white at room temperature). Furthermore, we tested if repetitive (cumulative) coagulation has an impact on the overall form and size of the clot.

**Findings:**

Coagulation-growth was achieved as a function of power and duration of coagulation; reproducible well-formed thermocoagulations could be achieved. When using two adjacent electrodes a power range between 1.25 Watt and 2.00 Watt resulted in homogenous ovoid coagula. After two minutes of coagulation the clots reached a maximum in size and further growth could not be achieved. It was also possible to increase the size of a preformed clot by repetitive coagulation either by increasing the power level or the duration of the coagulation process.

**Conclusions:**

We could show that it is possible to obtain predictable coagula in-vitro when using DBS electrodes for thermocoagulation even though they have not been developed for that specific purpose. However, until in-vivo safety and efficacy of DBS electrodes for ablation purposes is properly assessed, only approved electrodes should be used for brain ablation.

## Background

Today Deep Brain Stimulation (DBS) is state-of-the-art for treating various cases of movement disorders and it has widely replaced conventional therapeutic lesioning (thalamotomy, pallidotomy) [1]. The adaptability and reversibility of DBS makes it superior to ablative procedures. Tremor, rigidity, and dystonia can be efficiently treated. However, there are conditions where the therapy has to be discontinued. Complications such as infection or erosion, loss of effect, tolerance, pain or discomfort and device failure can result in terminating the DBS treatment [2]. Also financial limitations can be a reason for terminating the DBS treatment [3]. To us it seems feasible and practical to perform lesions using DBS electrodes which are already at the right location. However, it remained unclear whether these lesions are predictable in size and geometry, a key factor for a safe use of ablative procedures.

The aim of this study was to determine if it is possible to generate coagula with a predictable geometry and to define their dimensions as a function of power and time in an in vitro model (egg white at room temperature). Furthermore, we tested if repetitive coagulations could lead to a predictable change of size and shape of a pre-existent coagulum.

## Methods

**Figure 1:**
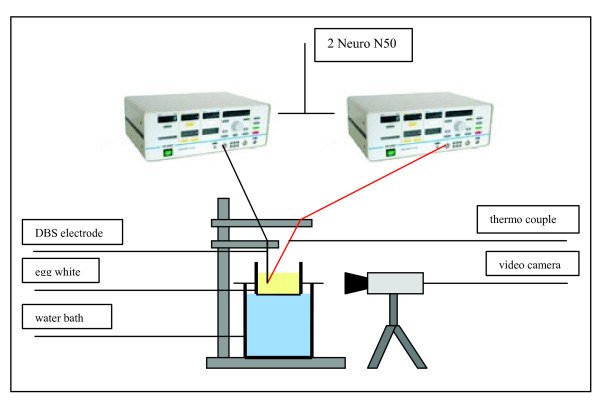
**Setup of the experiment**.

Our laboratory setup was similar to the ones described in [4-6]. In a container filled with egg white we slowly inserted the DBS probe. The slow insertion of the DBS probes into the egg white, which we kept at room temperature, prevented the formation of air bubbles leading to optimised visibility. A thin copper plate on the inner side of one of the flat walls of the container acted as reference electrode for monopolar coagulations. Thermocouples placed at a maximum distance of 1 mm next to the electrode were used to monitor temperature development (figure 1, table 1).

**Table 1:** Means and standard deviations of temperatures reached in function of lesioning duration and power.

	1.25 W	1.51 W	1.73 W	2.00 W
**30 s**	59.2 +/- 4.0°C	65.9 +/- 4.4°C	67.2 +/- 3.7°C	71.8 +/- 4.2°C
**60 s**	63.5 +/- 4.2°C	69.3 +/- 3.5°C	73.7 +/- 5.3°C	82.1 +/- 5.7°C
**90 s**	66.3 +/- 5.2°C	71.3 +/- 4.6°C	76.6 +/- 5.5°C	82.9 +/- 5.9°C
**120 s**	68.1 +/- 5.5°C	72.9 +/- 4.7°C	78.3 +/- 5.2°C	85.5 +/- 7.1°C

A 500 kHz radio frequency (RF) generator (Neuro N50, Stockert GmbH) delivering the high frequency current to the DBS electrodes was connected to a PC through an analogue/digital converter.

The software EPWIN-N50 (Stockert GmbH) controlled the ablation current and graphically displayed the electrode impedance, current and voltage over time. The ablation process was monitored using a digital video camera. Subsequently taken screenshots allowed to measure the coagulum size at different stages of its growth.

To compare the mean and standard deviation values of the coagulum growth we systematically increased the coagulation power and duration. For repeatability each combination of power and time was repeated five times in separate samples. To investigate coagulum growth of cumulative ablation, we performed a primary coagulation and after a short cooling period different parameters were used for another coagulation process. Pictures of the coagulum after standardized time points allowed measuring the coagulum size (the diameters of the probe and the lesion were measured in pixels in each picture and as the diameter of the probe is known, the size of the coagulum could be calculated).

## Findings

**Figure 2:**
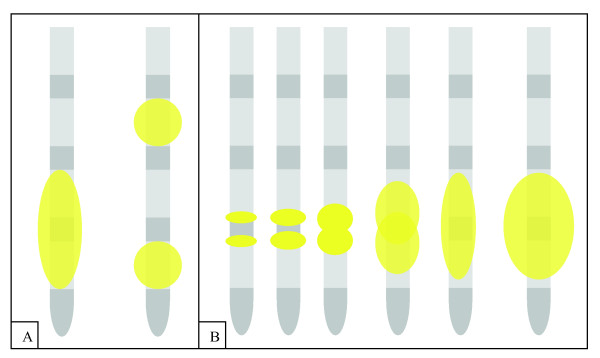
**Schematic illustration of lesion development**. a) Only when choosing two adjacent poles as active ablation electrodes homogenous coagulation ovoids formed. Ablation over distant poles resulted in two smaller single coagula with the same shape and size as monopolar coagulations. b) The origin of the resulting coagulations was situated near the two adjacent active poles of the quadripolar electrodes.

For the quadripolar electrodes we examined all six possible locations for bipolar ablation. However, only when choosing two adjacent poles as active ablation electrodes homogenous coagulation ovoids formed (figure 2a). The origin of the resulting coagulations was situated near the two adjacent active poles. Ablation over distant poles resulted in two smaller single coagula with the same shape and size as monopolar coagulations (figure 2b).

**Figure 3:**
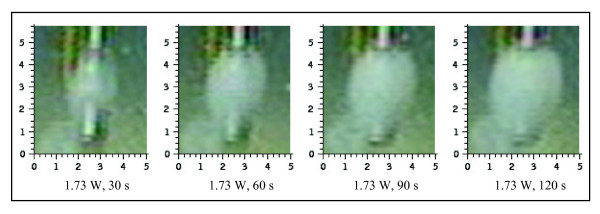
**Example of the development of a lesion obtained with 1.73 Watt over 120 seconds**. Homogenous ovoid lesions increased in size in function of lesioning duration.

**Figure 4:**
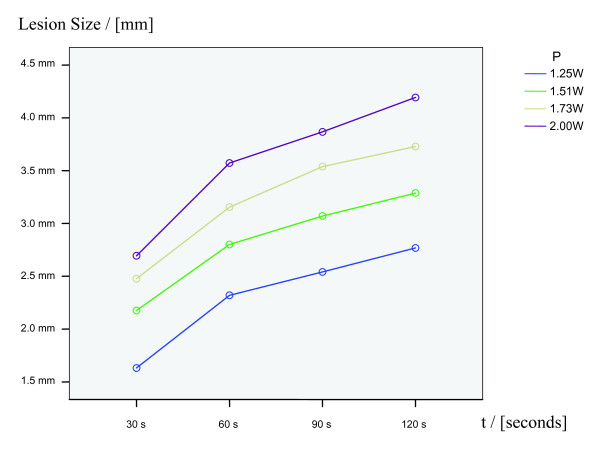
**Result of the variance analysis**. Lesion growth shows the trend to be as a function of time and/or duration.

Lesion size is as a function of power and duration. Increasing power and/or lesioning time showed the trend of resulting in larger coagula (figure 3 and 4). Predictable and reproducible results were obtained in the power range between 1.25 Watt and 2.00 Watt over the duration of a maximum of 120 seconds. With less power than 1.25 Watt no well-formed thermocoagulations could be obtained. Applying more than 2.00 Watt resulted in vaporisation and it was not possible to achieve homogenous coagula. The incremental increase in coagulation depth changed over time (figure 4). After two minutes of coagulation the clots reached a maximum size and further growth could not be achieved (plateau). The resulting clots were homogeneous and ovoid in shape.

Cumulative coagulation, in terms of increasing the size of a preformed coagulum, could be achieved. However, further coagulum growth could only be managed either by coagulating longer or with more power. For example, a coagulum generated with 1.25 Watt over 60 seconds could either be increased with 1.51 Watt over 60 seconds or with 1.25 Watt over the duration of more than 60 seconds. Applying the same parameters as the primary clot has been formed with - in this example 1.25 Watt over 60 seconds - did not lead to an increase in size. Table 2 summarizes the coagula's sizes for different power levels and coagulation durations.

**Table 2:** Means and standard deviations of lesions' maximum diameter depending on power and duration.

	1.25 W	1.51 W	1.73 W	2.00 W
**30 s**	1.63 +/- 0.67 mm	2.18 +/- 0.39 mm	2.48 +/- 0.34 mm	2.69 +/- 0.29 mm
**60 s**	2.32 +/- 0.42 mm	2.80 +/- 0.46 mm	3.15 +/- 0.51 mm	3.57 +/- 0.20 mm
**90 s**	2.54 +/- 0.32 mm	3.07 +/- 0.52 mm	3.54 +/- 0.58 mm	3.87 +/- 0.34 mm
**120 s**	2.78 +/- 0.36 mm	3.29 +/- 0.55 mm	3.73 +/- 0.72 mm	4.19 +/- 0.46 mm

## Discussion

There are conditions where patients treated with chronic stimulation discover long-term complications such as infection or erosion, loss of effect, tolerance, pain or discomfort. In these cases the therapy has to be discontinued and alternatives have to be found. We tested if it was possible to perform reproducible lesions with the already implanted and well-located DBS electrodes. We could show in our in-vitro egg white experiments that DBS electrodes can be used to form predictable, reproducible and homogeneous ovoid coagula. Due to a limited number of coagulations, we could only show the trend of their increase of size in function of power and/or lesioning duration.

The incremental increase in coagulation size reached a maximum after two minutes. We assume that various non-linear parameters are causing this effect. Change of electric properties due to increasing impedance caused by continuous tissue damage could be one explanation. Certain tissue properties, such as relative density and electrical conductivity, also vary in different anatomical structures (e.g. grey and white matter of the brain). Their impact on lesion size and shape should be observed by modern finite element methods. Although it has been stated that lesions' sizes obtained in egg white models are correlating with those obtained in vivo [6], a generalization of this method to neural tissue could be limited. Thus, to fully understand the efficacy and safety of using DBS electrodes for ablation purposes, the comparison of in vitro, computational (FEM) and in vivo experiments appear to be mandatory. At this point in time it is important to mention that these electrodes are not approved for ablation procedures and only approved electrodes should be used for brain ablation.

Next steps in our research are to use computational prediction of thermo ablation procedures in homogeneous media and then in anatomically correct models of the human anatomy. These predictions will include non-linear tissues parameters and account for blood flow. Using these novel techniques we hope to develop ablation electrodes for various coagula shapes using multiple electrodes and computer controlled ablation parameters.

## Conclusion

Using DBS electrodes it is possible to produce reproducible and homogeneous thermo-coagulations with predictable size and shape in egg white.

## Competing interests

The authors declare that they have no competing interests.

## Authors' contributions

FA conceived of the study, and participated in its design and coordination and helped to draft the manuscript. TLH carried out the experiments and performed the statistical analysis. HL participated in the study's design and helped with technical and physical questions. WK participated in the study's design and revised the results critically.

All authors read and approved the final manuscript.
